# Association between total prehospital time and early mortality in patients with severe torso trauma: a retrospective study at an urban Emergency and Critical Care Center

**DOI:** 10.1186/s12245-026-01249-3

**Published:** 2026-05-08

**Authors:** Megumi Iwasaki, Mitsuaki Kojima, Yuzuru Mochida, Raira Nakamoto, Tomohisa Shoko

**Affiliations:** Department of Emergency and Critical Care Medicine, Tokyo Women’s Medical University Adachi Medical Center, Adachi City, Tokyo Japan

**Keywords:** Severe torso trauma, Total prehospital time, Early mortality, Urban trauma system, Geographic information system

## Abstract

**Background:**

In urban emergency and critical care centers (ECCCs), transport distances are relatively short, and severely injured trauma patients may reach the hospital before cardiac arrest occurs. Severe torso trauma is relevant as it may involve time-sensitive hemorrhage in the chest, abdomen, or pelvis. However, the association between total prehospital time (TPT) and early mortality has not been well examined in urban settings in Japan. We aimed to investigate this relationship in patients with severe torso trauma treated at an urban ECCC.

**Methods:**

This single-center retrospective study was conducted at an urban ECCC in Tokyo from March 2016 to December 2024. We reviewed data from 342 patients with an Abbreviated Injury Scale score ≥ 3 for the neck, chest, abdomen, or pelvis. Associations between TPT and death within 24 h, Injury Severity Score (ISS), Revised Trauma Score (RTS), and probability of survival (P_s_) were assessed. Geographic Information System was used to evaluate spatial patterns, and Delta Shock Index (DSI) was used to assess hemodynamic changes during transport. Patients meeting predefined exclusion criteria were excluded.

**Results:**

Of the 342 patients, 20 patients died within 24 h of arrival, and no deaths were observed in patients with a TPT ≥ 61 min. Linear regression analysis revealed significant associations between shorter TPT and severity indicators (ISS, RTS, and P_s_). Logistic regression analysis showed a significant association between ISS and early mortality. TPT was not significantly associated with early mortality after adjusting for ISS. Of the early deaths, 75% occurred within 5 km of the ECCC. Although no significant difference in DSI was observed between survivors and non-survivors, patients with an ISS of 25–49 and a DSI ≥ 0.1 had shorter TPT.

**Conclusion:**

In this urban trauma system, shorter TPT was associated with higher early mortality. This may reflect rapid transport of critically injured patients rather than a harmful effect of shorter TPT itself. In settings with relatively short prehospital times, this association should be interpreted in the context of trauma severity and trauma system structure, and optimizing early in-hospital trauma response may be more important than further reducing transport time.

**Supplementary Information:**

The online version contains supplementary material available at 10.1186/s12245-026-01249-3.

## Introduction

Time is an important factor that affects the prognosis of trauma patients [1–8]. Based on established principles [9–11] and empirical evidence [12], shorter transport time improves outcomes. Organized trauma systems aim to shorten prehospital time, based on the assumption that rapid transfer to a trauma center improves the survival and recovery rate [1, 13, 14]. Within trauma systems, patient outcomes are influenced not only by prehospital time but also by whether patients are transported to facilities capable of providing appropriate level of definitive trauma care. In systems such as that in Japan, where prehospital interventions are relatively limited and physician-staffed response units are not routinely available across all regions, the effectiveness of trauma care relies heavily on rapid transport and appropriate hospital destination selection. Therefore, understanding how total prehospital time (TPT) affects outcomes in such systems is important. However, some studies have found no significant association between prehospital time and mortality [4, 15–19], suggesting that reduced prehospital time may not uniformly improve outcomes.

Urban areas tend to have shorter prehospital times, compared with rural regions [20–23], and the emergency and critical care centers (ECCCs) in Tokyo are characterized by short transport distances and high-volume patient intake. This study was conducted at an urban ECCC in a densely populated part of Tokyo, where most trauma patients are transported within 1 h of injury. The proximity of medical facilities and dense emergency coverage means that severely injured patients are often transported before cardiac arrest occurs. This shorter TPT group is likely to include patients with poor prognoses due to critical injury severity, potentially introducing bias into the observed association. This bias, known as the “scoop-and-run bias” [24], refers to the preferential transport of critically injured patients, resulting in higher mortality rates in the shorter TPT group.

Studies have indicated the association of shorter prehospital times with increased mortality [25–27], particularly among patients with severe torso or blunt trauma injuries, with the risk of death peaking within the first 30 min [25, 28]. These findings underscore the importance of early medical intervention; however, the structure and confounding influences surrounding the association between TPT, defined as the interval from emergency medical service (EMS) dispatch to hospital arrival, and early mortality in urban ECCCs in Japan remain insufficiently investigated. In this study, we focused on severe torso trauma involving the chest, abdomen, or pelvis with AIS score ≥ 3 because these injuries may involve life-threatening, time-sensitive hemorrhage, and rapid assessment of the bleeding source and severity is often difficult in the prehospital setting. In contrast, hemorrhage from many extremity injuries can often be controlled prehospital with tourniquet use, whereas in severe traumatic brain injury, prognosis is more directly determined by primary injury and may be less closely related to prehospital transport time. Therefore, we considered patients with severe torso trauma a clinically important population for examining the association between TPT and early outcomes. Although several studies have examined the association between prehospital time and trauma outcomes, relatively few studies have specifically focused on patients with severe torso trauma.

In this study, TPT was treated as a non-modifiable factor reflecting patient condition at hospital arrival. We hypothesized that shorter TPT would be associated with higher early mortality in urban trauma systems because severely injured patients are transported more rapidly to trauma centers.

Therefore, we aimed to examine the association between TPT and early mortality within 24 h of hospital arrival in patients with severe torso trauma transported to an urban ECCC. We also explored the relationship between TPT, injury severity, and geographic distribution of injury events in an urban EMS system.

## Methods

### Study setting

This retrospective observational study was conducted at a single ECCC in the Tokyo metropolitan area (see Additional File 1 for details). Of 1,297 trauma patients transported to our center between March 1, 2016 and December 31, 2024, those with an Abbreviated Injury Scale (AIS) score ≥ 3 for the neck, chest, abdomen, or pelvis were included. The AIS system classifies the type and anatomical severity of trauma on a 6-point scale. This study focused on severe torso trauma involving the neck, chest, abdomen, or pelvis, where life-threatening hemorrhage is a major concern, including cervical vascular injuries that may lead to hemorrhagic shock. Patients with concomitant traumatic brain injury (TBI) were not excluded if they met the inclusion criteria for severe torso trauma. Patients who developed cardiac arrest during transport or upon hospital arrival were included in the analysis, whereas those experienced had cardiac arrest at the scene were excluded. Resuscitative efforts by EMS personnel had been performed in these cases as well.

The Tokyo emergency medical service (EMS) system is a fire department-based system in which patients are transported to appropriate hospitals based on injury severity and hospital availability.

Patients with AIS score < 3, those younger than 16 years, those who had cardiac arrest at the scene, those transferred from other hospitals, those who received on-site medical intervention by the disaster medical assistance team (DMAT), those involved in rescue operations lasting more than 1 h at the scene, those upgraded to tertiary transport due to coordination difficulties, patients in whom hemorrhagic death was not supported by postmortem imaging, and those with trauma mechanisms other than blunt or penetrating injury (e.g., burns or electrical injuries) were excluded.

In this study, analysis focused on hemorrhagic shock, which is more likely to be influenced by TPT. Therefore, cases in which hemorrhagic shock due to torso injury was not supported as the primary cause of death based on postmortem imaging and clinical records were excluded. Cases were not excluded solely because non-hemorrhagic findings were present as contributory factors.

Patients who received advanced on-site medical interventions, such as airway management, circulatory support, or procedural treatment by DMAT, were excluded to minimize the influence of prehospital medical interventions on the interpretation of TPT. In Japan, prehospital endotracheal intubation for trauma patients by EMS personnel is extremely rare and typically occurs only in the context of advanced on-site interventions provided by physician-staffed teams or DMAT.

Further details regarding the Tokyo trauma system structure, EMS system, and DMAT are provided in the Supplementary Methods.

This study was conducted in accordance with the Declaration of Helsinki and approved by the Institutional Review Board of Tokyo Women’s Medical University (approval no. 2023 − 0207). As the study involved no direct intervention and utilized only anonymized clinical data, written informed consent was waived. Information regarding study purpose and content was disclosed on the hospital bulletin board and website, and an opt-out process was provided to ensure patient autonomy.

### Data collection

Age, sex, prehospital details (EMS dispatch location, mechanism of injury, and times of EMS call, arrival at the scene, departure from the scene, and hospital arrival), vital signs before hospital arrival (blood pressure, heart rate, respiratory rate, and level of consciousness assessed using the Japan Coma Scale [JCS]), vital signs when arriving at the emergency department (ED) (blood pressure, heart rate, respiratory rate, and Glasgow Coma Scale [GCS] score), and occurrence or non-occurrence of early death within 24 h of hospital arrival were extracted from medical records, whereas trauma classification, physiological severity based on Revised Trauma Score (RTS), anatomical severity based on Injury Severity Score (ISS), and probability of survival (P_s_, based on age-adjusted physiological and anatomical parameters using Trauma and Injury Severity Score [TRISS]) were obtained from data registered by our center at the Japan Trauma Data Bank (JTDB). RTS was calculated using systolic blood pressure, respiratory rate, and GCS score recorded at ED arrival, whereas ISS was calculated according to the 2005 version of AIS (updated in 2008). Further details regarding the TRISS methodology are provided in the Supplementary Methods. The JTDB, established in 2003 and jointly operated by the Japan Association for Trauma Surgery and Japanese Association for Acute Medicine, is a nationwide trauma registry that collects standardized data on trauma patients with an Abbreviated Injury Scale (AIS) score ≥ 3.

### Outcome measurement

In the analysis, TPT was treated as a non-modifiable variable reflecting patient condition at hospital arrival.

The primary outcome, early death, was defined as death within 24 h of hospital arrival. This threshold is commonly used in trauma research because most hemorrhage-related deaths occur within the first 24 h post-injury, which is considered to reflect the time during which delays in prehospital care and definitive hemorrhage control may have the greatest impact on outcomes.

**Fig. 1:**
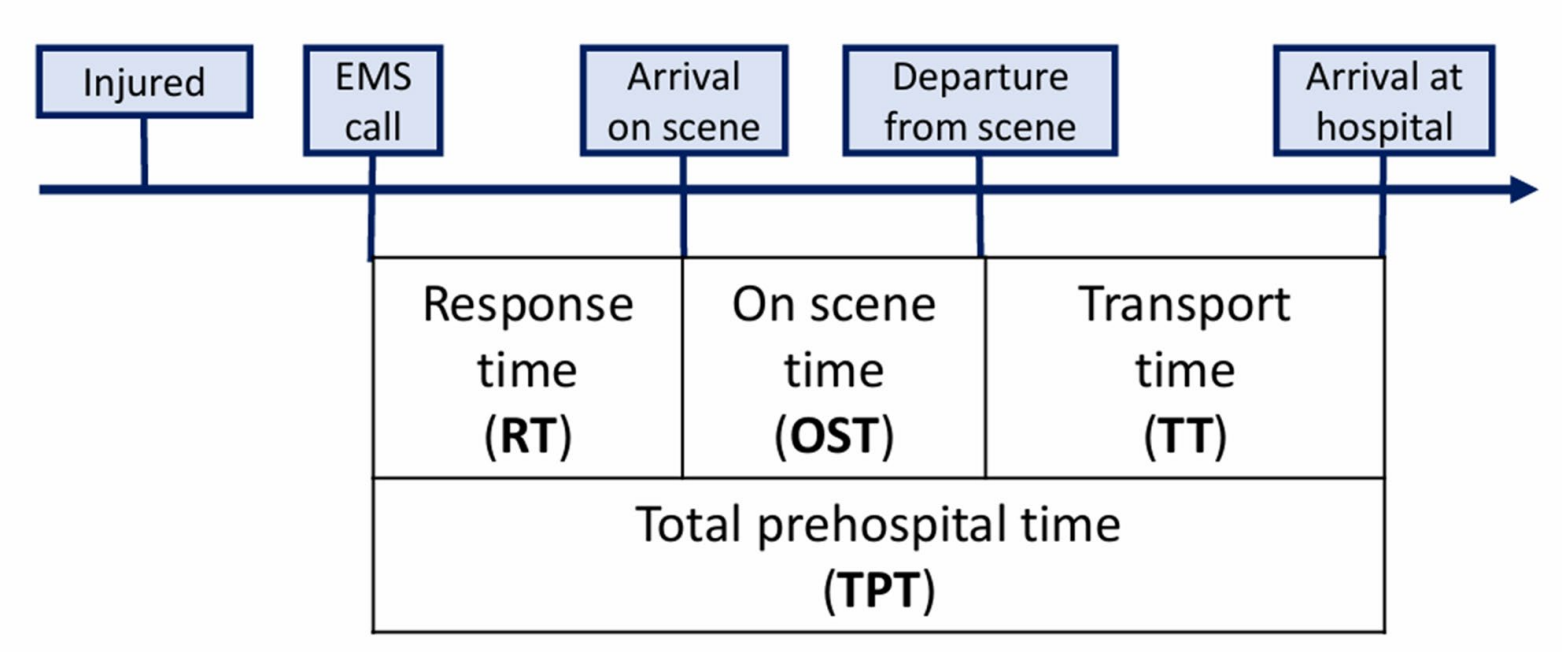
Definition of total prehospital time (TPT) in trauma care. The figure illustrates the sequential phases of prehospital management—response time (RT), on-scene time (OST), and transport time (TT)—which together constitute TPT

The association between TPT, defined as the interval between EMS call and hospital arrival, and early mortality was analyzed according to three TPT components: the response time (RT; time from EMS call to arrival at the scene), on-scene time (OST; time from arrival to departure from the scene), and transport time (TT; time from scene departure to hospital arrival) (Fig. 1). TPT intervals were set at 15-minute increments and patients with TPT < 15 min were excluded due to insufficient sample size.

The secondary outcome, Delta Shock Index (DSI), assessed changes in patient physiology before and after transport. Shock index (SI) was calculated as heart rate divided by systolic blood pressure. SI on ED arrival was calculated only in patients with measurable vital signs on arrival; patients who were dead on arrival (DOA) were excluded from this calculation. Hemorrhagic shock was defined as an SI ≥ 1.0 on ED arrival, based on previous trauma studies [29, 30].

DSI was defined as SI on ED arrival minus that at the scene. A threshold of DSI > 0.1 was used based on previous studies [31, 32]. A time-normalized version of DSI, defined as DSI divided by TPT in minutes, was also utilized to evaluate the rate of physiological deterioration.

The geographic distribution of patients was analyzed using prehospital SI and stratified into four groups based on prior studies [29, 30]: no shock (SI < 0.5), mild shock (SI of 0.5–1.0), moderate shock (SI of 1.0–1.5), or severe shock (SI ≥ 1.5). Transport distance was calculated using the Manhattan distance, derived from geocoded EMS dispatch and hospital location (latitude and longitude). As the hospital relocated by approximately 2.84 km on January 1, 2022, transport distances were adjusted to reflect pre- and post-relocation coordinates in accordance with hospital arrival date. Patient geographic distribution was visualized using QGIS version 3.32 (QGIS Developer Team, Lima 2023).

### Statistical analysis

All statistical analyses were performed using R version 3.6.1 (The R Foundation for Statistical Computing, Vienna, Austria) and EZR (Saitama Medical Center, Jichi Medical University, Saitama, Japan) [33].

Continuous variables with non-normal distribution were presented as medians with interquartile ranges (IQRs) and compared using the Mann–Whitney U test. Categorical variables were expressed as percentages and compared using Fisher’s exact test. The primary outcome was early death, defined as death within 24 h of hospital arrival. To evaluate the association between early mortality and explanatory variables, logistic regression analysis was performed using TPT and ISS as covariates. Early death occurred in only 20 patients; therefore, to avoid overfitting and maintain appropriate balance between the number of events and predictors, the regression model was restricted to minimal covariates [34]. Variables were selected a priori based on clinical relevance and previous literature.

To investigate the quantitative association between TPT and trauma severity, separate linear regression analyses were conducted with ISS, RTS, and P_s_ as dependent variables. Sensitivity analyses were also conducted; however, patients confirmed DOA were excluded to minimize confounding. All statistical tests were two-sided, and a p-value < 0.05 was considered statistically significant.

## Results

### Patient selection and exclusion criteria

**Fig. 2:**
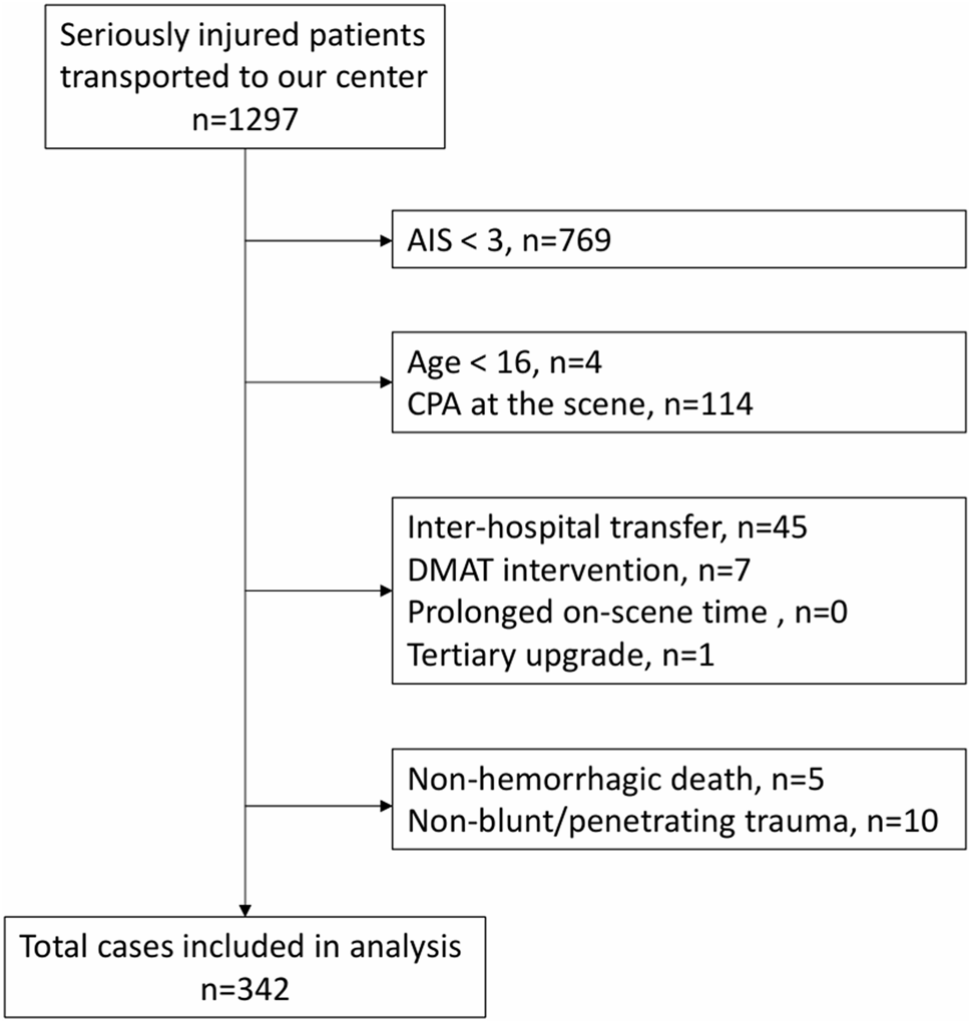
Flowchart of patient selection for analysis. Among 1,297 trauma patients transported to the center, exclusion criteria were applied—including AIS score < 3, age < 16 years, cardiopulmonary arrest (CPA) at the scene, inter-hospital transfer, disaster medical assistance team (DMAT) intervention, and other factors—resulting in 342 patients included in the final analysis

Overall, 1,297 trauma patients were transported to our center during the study period; based on the exclusion criteria, 955 were excluded and 342 were included in the final analysis (Fig. 2). None met the exclusion criteria related to prolonged rescue operations, defined as an OST ≥ 60 min.

### Baseline characteristics of patients

Baseline characteristics of the patients are summarized in Table 1. Their median age was 55.5 (IQR: 41.0–71.0) years, and 75.7% were male (259/342). Blunt trauma accounted for 89.2% of the total cases (305/342). The most common injury mechanisms were falls or falls from height (46.6%, 142/342), followed by motor vehicle accidents (44.9%, 137/342). Penetrating trauma accounted for 10.8% (37/342) and was mainly related to stab injuries.

**Table 1:** Patient characteristics in the study groups

		Category						*p*-value
		Total (*n* = 342)		Early death group (*n* = 20)		Survival group (*n* = 322)		
Demographics								
	Sex, male, n (%)	259	(75.7%)	19	(95.0%)	240	(74.5%)	0.055
	Age, median (IQR)	55.5	(41.0–71.0)	55.5	(47.3–69.3)	55.5	(41.0–71.8)	0.931
Type of trauma								
	Blunt, n (%)	305	(89.2%)	16	(80.0%)	289	(89.8%)	0.252
	Penetrating, n (%)	37	(10.8%)	4	(20.0%)	33	(10.2%)	0.252
Mechanism of injury								
	Traffic accident, n (%)	137	(40.1%)	5	(25.0%)	132	(41.0%)	0.239
	Fall, n (%)	142	(41.5%)	11	(55.0%)	131	(40.7%)	0.245
	External force, n (%)	47	(13.7%)	4	(20.0%)	43	(13.4%)	0.498
	Other, n (%)	16	(4.7%)	0	(0.0%)	16	(5.0%)	0.611
Prehospital time								
	TPT, min, median (IQR)	37.0	(32.0–46.0)	36.0	(24.8–40.3)	38.0	(32.0–46.0)	0.093
	TPT 16–30, n (%)	61	(17.8%)	6	(30.0%)	55	(17.1%)	0.142
	TPT 31–45, n (%)	189	(55.0%)	12	(60.0%)	176	(54.7%)	0.818
	TPT 46–60, n (%)	81	(23.7%)	2	(10.0%)	79	(24.5%)	0.179
	TPT ≥ 61, n (%)	12	(3.5%)	0	(0.0%)	12	(3.7%)	1.00
	RT, min, median (IQR)	8.0	(6.0–11.0)	6.0	(5.0–9.5)	8.0	(6.0–11.0)	0.089
	OST, min, median (IQR)	15.0	(11.0–21.0)	15.0	(10.8–20.3)	15.0	(11.3–21.0)	0.588
	TT, min, median (IQR)	13.0	(10.0–18.0)	11.5	(8.8–15.0)	13.0	(10.0–18.0)	0.112
Transport distance								
	Distance, km, median (IQR)	4.4	(2.8–6.3)	4.1	(2.6–5.0)	4.4	(2.8–6.4)	0.332
Physiology at scene								
	SI at scene, median (IQR)	0.80	(0.61–1.11)	1.41	(1.12–2.31)	0.79	(0.60–1.03)	< 0.001
Physiology on ED arrival								
	Measurable vital signs on ED arrival, n (%)	337	(98.5%)	15	(75.0%)	322	(100.0%)	< 0.001
	SI on ED arrival, median (IQR)	0.68	(0.55–0.92)	1.53	(0.89–2.35)	0.67	(0.54–0.889)	< 0.001
	Hemorrhagic shock (SI ≥ 1.0 on ED arrival), n (%)	69	(20.5%)	10	(66.7%)	59	(18.3%)	0.002
DSI								
	DSI ≥ 0.1, n (%)	60	(17.5%)	5	(25.0%)	55	(17.1%)	0.366
Injury severity								
ISS, median (IQR)		17	(10–25)	29	(21–35)	17	(10–25)	< 0.001
	ISS ≥ 50, n (%)	6	(1.8%)	2	(15.0%)	4	(1.2%)	0.042
	ISS 25–49, n (%)	101	(29.5%)	12	(60.0%)	89	(27.6%)	0.004
	ISS 16–24, n (%)	108	(31.6%)	4	(20.0%)	104	(32.3%)	0.326
	ISS < 16, n (%)	127	(37.1%)	2	(10.0%)	125	(38.8%)	0.008
RTS, median (IQR)		7.8	(6.9–7.8)	1.9	(0.5–5.6)	7.8	(7.1–7.8)	< 0.001
	RTS < 2.0, n (%)	12	(3.5%)	11	(55.0%)	1	(0.3%)	< 0.001
	RTS 2.0–4.0, n (%)	4	(1.2%)	2	(10.0%)	2	(0.6%)	0.018
	RTS 4.0–6.0, n (%)	45	(13.2%)	3	(15.0%)	42	(13.0%)	0.736
	RTS ≥ 6.0, n (%)	281	(82.2%)	4	(20.0%)	277	(86.0%)	< 0.001
P_s_, median (IQR)		0.95	(0.87–0.98)	0.20	(0.02–0.62)	0.96	(0.89–0.98)	< 0.001
	P_s_ < 0.25, n (%)	15	(4.4%)	11	(55.0%)	4	(1.2%)	< 0.001
	P_s_ 0.25–0.5, n (%)	18	(5.3%)	2	(10.0%)	16	(5.0%)	0.284
	P_s_ ≥ 0.5, n (%)	309	(90.4%)	7	(35.0%)	302	(93.8%)	< 0.001
Emergency procedures								
	MTP, n (%)	60	(17.5%)	18	(90.0%)	42	(13.0%)	< 0.001
	ERT, n (%)	23	(6.7%)	14	(70.0%)	9	(2.8%)	< 0.001
	Blood transfusion within 24 h, n (%)	126	(36.8%)	18	(90.0%)	108	(33.5%)	< 0.001
	RBC transfusion within 24 h, units, median (IQR)	0	(0–6)	15	(9-24.5)	0	(0–4)	< 0.001
	Emergency surgery, n (%)	152	(44.4%)	4	(20.0%)	148	(46.0%)	< 0.001
	Damage control surgery, n (%)	24	(7.0%)	9	(45.0%)	15	(4.7%)	< 0.001
	IVR performed, n (%)	63	(18.4%)	4	(20.0%)	59	(18.3%)	0.772

IQR, interquartile range; TPT, total prehospital time; ED, emergency department; RT, response time, OST, on-scene time; TT, transport time; SI, shock index; DSI, Delta Shock Index; ISS, Injury Severity Score; RTS, Revised Trauma Score; P_s_, probability of survival; MTP, massive transfusion protocol; ERT, emergency resuscitative thoracotomy; RBC, red blood cell; IVR, interventional radiology

*Hemorrhagic shock was defined as SI ≥ 1.0 on ED arrival and was calculated only among patients with measurable vital signs on ED arrival (*n* = 337)

The median ISS was 17 (IQR: 10–25), and the median P_s_ calculated using TRISS was 0.95 (IQR: 0.87–0.98). Additional clinical interventions and physiological variables are presented in Table 1. SI at the scene and on ED arrival were significantly higher in the early-death group versus the survival group, indicating more severe hemodynamic instability.

Hemorrhagic shock, defined as an SI ≥ 1.0 on ED arrival, was significantly more frequent in the early death group versus the survival group (66.7% vs. 18.3%; *p* < 0.001).

Massive transfusion protocol activation, emergency resuscitative thoracotomy, blood transfusion within 24 h, and damage control surgery were significantly more common in the early-death group, whereas emergency surgery was less frequently performed in the early-death group. Interventional radiology was not significantly different between the groups. 

### Primary outcome: early mortality and associated factors

Early death occurred in 20 patients (5.8%). The median ISS among the deceased patients was 29 (IQR: 21–35). Five patients were DOA. Overall, 23 patients underwent resuscitative thoracotomy during the study period. Of the 15 post-arrival cardiac arrest cases, the median time from hospital arrival to initiation of cardiopulmonary resuscitation was 27.0 (IQR: 12.3–51.8) minutes. Comparisons of the early-death and survival groups revealed significant differences in P_s_, ISS, RTS, and SI, both prehospital and upon ED arrival (all *p* < 0.001). We used a logistic regression model adjusted for ISS to assess the association between TPT and early mortality, considering the limited number of events.

### TPT: mortality trends and injury severity

The median TPT was 37.0 (IQR: 32.0–46.0) minutes, with values of 36.0 (IQR: 24.8–40.3) and 38.0 (IQR: 32.0–46.0) minutes in the early-death and survival groups, respectively. The median RT, OST, and TT were 8.0 (IQR: 6.0–11.0), 15.0 min (IQR: 11.0–21.0), and 13.0 (IQR: 10.0–18.0) minutes, respectively.

**Fig. 3:**
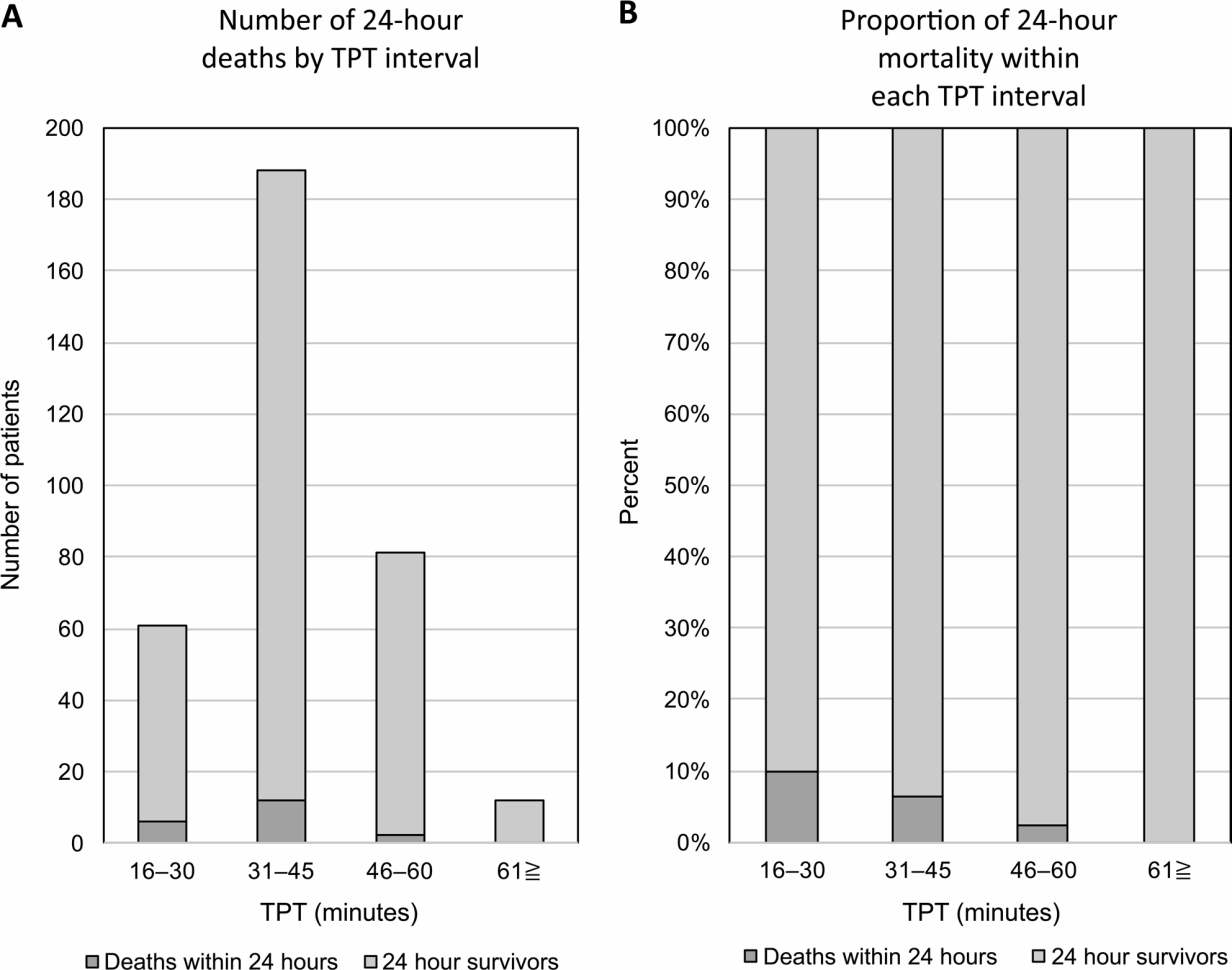
Relationship between total prehospital time (TPT) and 24-hour mortality. (**A**) Number of deaths and survivors within each TPT interval. (**B**) Proportion of 24-hour mortality across TPT intervals, showing higher mortality in shorter TPT groups, likely reflecting prioritization of severely injured patients for rapid transport

**Fig. 4:**
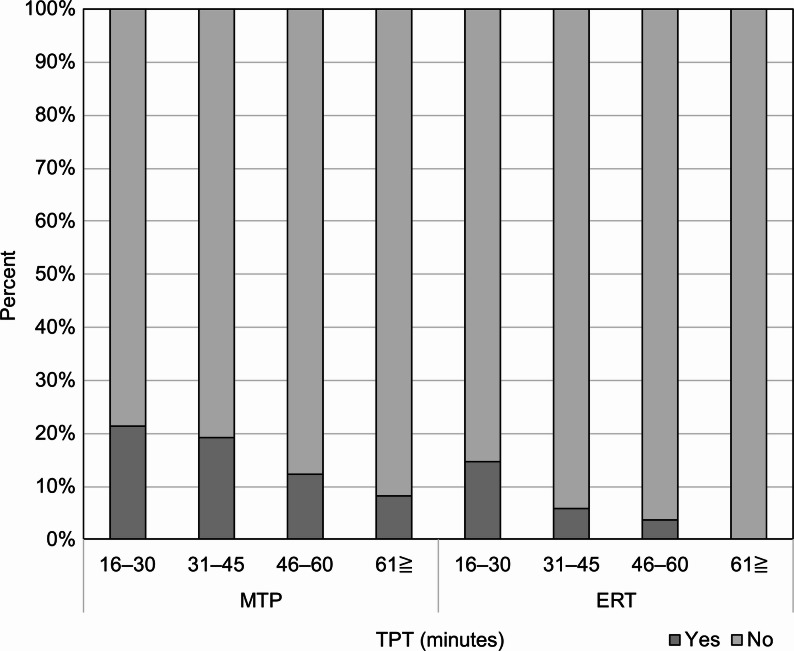
Massive transfusion protocol (MTP) and emergency resuscitative thoracotomy (ERT) across total prehospital time (TPT) intervals. Higher rates of MTP and ERT are observed in shorter TPT groups, suggesting that patients with greater injury severity are more frequently represented in these intervals

Early mortality varied by TPT category, with 9.8% (6/61), 6.4% (12/188), and 2.5% (2/81) rates observed for patients with TPTs of 16–30, 31–45, and 46–60 min, respectively. No early deaths were observed in patients with a TPT ≥ 61 min (Fig. 3A and B). A shorter TPT was associated with higher rates of emergency interventions, including massive transfusion protocol activation and emergency resuscitative thoracotomy (Fig. 4).

**Fig. 5:**
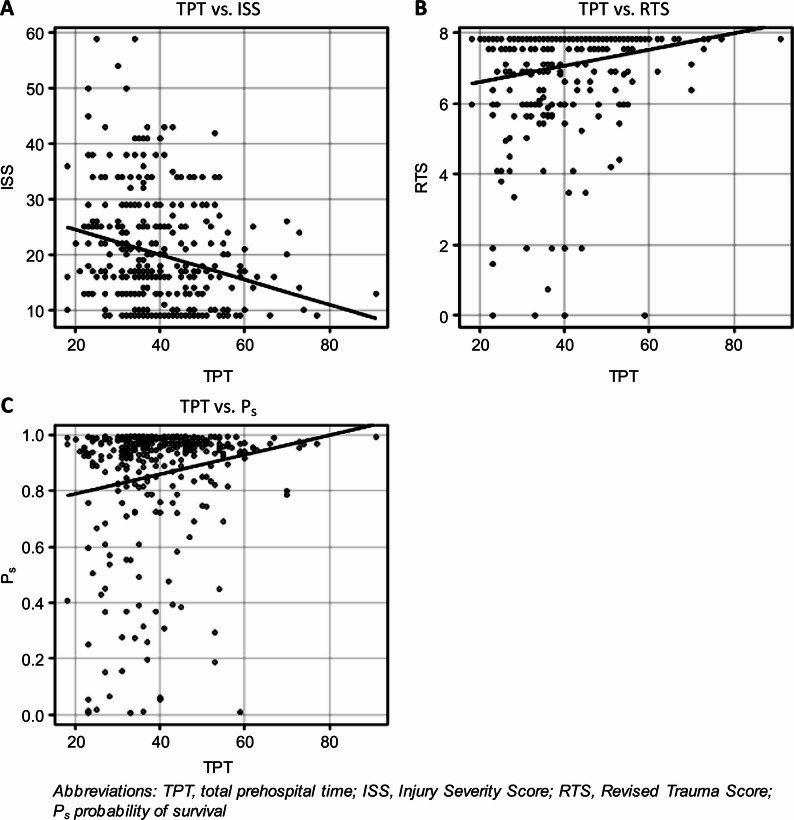
Association between total prehospital time (TPT) and severity indicators evaluated using linear regression. (**A**) TPT vs. Injury Severity Score (ISS): a significant negative correlation (β = − 0.225, *p* < 0.001), indicating that shorter TPT is associated with higher ISS. (**B**) TPT vs. Revised Trauma Score (RTS): a weak positive correlation (β = 0.023, *p* = 0.0018), suggesting that longer TPT is associated with more stable physiological status. (**C**) TPT vs. probability of survival (P_s_): a positive correlation (β = 8.26, *p* = 0.0017), indicating that patients with longer TPT have a higher predicted survival rate

To evaluate the quantitative association between TPT and trauma severity, linear regression analyses were conducted using ISS, RTS, and P_s_ as dependent variables. A significant negative association was found between TPT and ISS (β = − 0.225; 95% confidence interval [CI]: − 0.322 to − 0.129; *p* < 0.001; Fig. 5A), indicating association of a shorter TPT with higher injury severity. Conversely, significant positive associations of TPT were observed with RTS (β = 0.023; 95% CI: 0.009 to 0.038; *p* = 0.0018; Fig. 5B) and P_s_ (β = 8.26; 95% CI: 3.13 to 13.38; *p* = 0.0017; Fig. 5C).

Based on the observed trends in severity and poor prognosis, logistic regression analysis was conducted to evaluate the association between TPT and early mortality, adjusting for ISS. The results showed a significant association between ISS and early mortality (odds ratio [OR]: 1.070; 95% CI: 1.030–1.110; *p* = 0.001), indicating an association between higher injury severity and increased risk of early death. In contrast, no significant association was observed between TPT and early mortality in the ISS-adjusted model (OR: 0.975; 95% CI: 0.926–1.030; *p* = 0.322).

Sensitivity analysis excluding patients confirmed DOA revealed a significant association between ISS and early mortality (OR: 1.08; 95% CI: 1.03–1.13; *p* < 0.001). However, no significant association was found between TPT and early mortality (OR: 0.97; 95% CI: 0.91–1.03; *p* = 0.339). The complementary analysis showed consistent results, with ISS remaining a significant predictor of early death (OR: 1.070; 95% CI: 1.020–1.130; *p* = 0.004) and the association with TPT lacking statistical significance (OR: 0.991; 95% CI: 0.929–1.060; *p* = 0.779).

### Geospatial analysis and transport distance

**Fig. 6:**
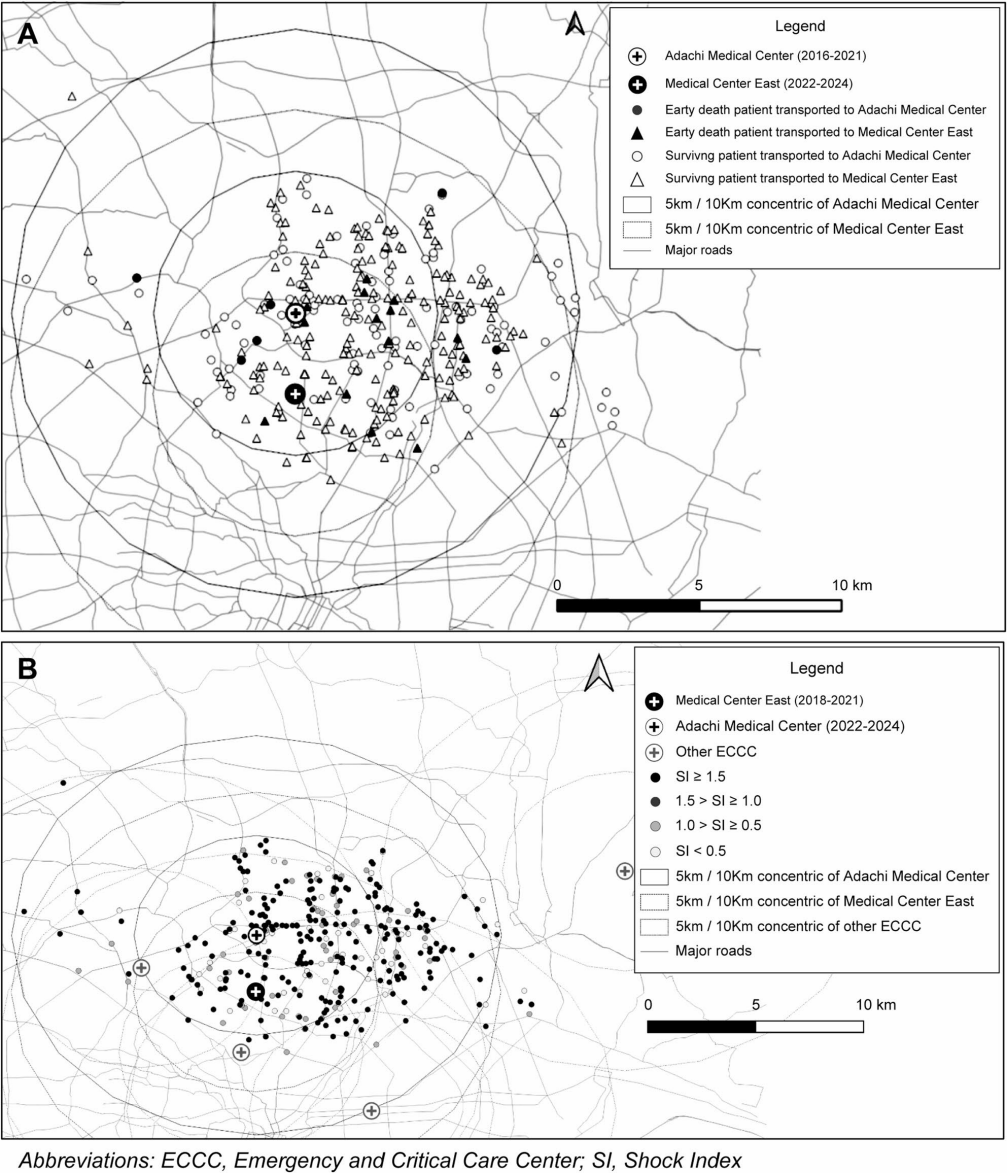
Geographic distribution of trauma patients, early mortality, and physiological severity. (**A**) Spatial distribution of location of trauma patients and early mortality. (**B**) Geographic distribution of physiological severity based on the shock index (SI) measured at the scene. Each marker represents one patient, color-coded by outcome or severity level

No significant intergroup difference was observed in terms of transport distance (*p* = 0.332), and geospatial mapping using GIS revealed no evidence of trauma case clustering, such as traffic accidents or falls at specific locations. The patient origin points in relation to the pre- and post-relocation hospital locations are visualized in Fig. 6A, together with the early death status. Notably, 75.0% of early deaths (15/20) occurred in patients transported from within a 5-km radius.

To assess any trends in injury severity by geographic location, prehospital SI derived from vital signs measured at the scene were mapped using GIS alongside patient origin data (Fig. 6B). Although this figure does not distinguish old and new hospital sites, severe trauma cases were relatively uncommon among patients located beyond the 5-km concentric zone.

### Secondary outcome: DSI and temporal variation

**Fig. 7:**
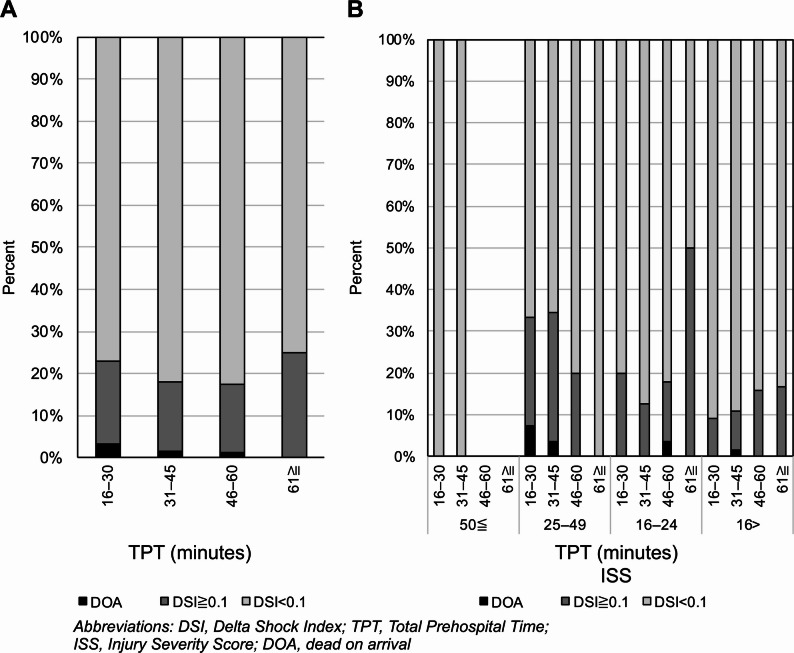
Delta Shock Index (DSI) ≥ 0.1 across total prehospital time (TPT) intervals. (**A**) Overall distribution of DSI ≥ 0.1 by TPT category. (**B**) Distribution of DSI ≥ 0.1 stratified by Injury Severity Score (ISS) and TPT category

Secondary outcome analysis using DSI revealed no significant intergroup differences (*p* = 0.909). Similarly, no significant association was observed between any TPT category and DSI (*p* = 0.986). The time-normalized DSI analysis, in which DSI was divided by time period in minutes to reflect time variation, yielded no significant difference (*p* = 0.991). However, distribution trends showed that patients with elevated DSI (≥ 0.1) were most frequently observed in the longest TPT group (≥ 61 min), followed by the shortest TPT group (16–30 min) (Fig. 7A). Additionally, stratified analysis using ISS and TPT categories revealed shorter TPT intervals for a higher proportion of patients in the group with ISS of 25–49 (Fig. 7B). 

The proportion of DOA patients was higher in the shortest TPT group, whereas that of patients with physiological deterioration during transport (DSI ≥ 0.1) was higher in the longer TPT group.

## Discussion

This study investigated the association between TPT and early mortality in patients with severe torso trauma transported to an urban ECCC. Severe torso trauma is clinically important as it may involve time-sensitive hemorrhage in the chest, abdomen, or pelvis. We selected 24-hour mortality as the primary outcome as it reflects the interval during which delays in prehospital care and hemorrhage control are most likely to influence patient outcomes, consistent with prior trauma literature.

In this study, a shorter TPT was associated with a higher proportion of early deaths. Patients in the short TPT group tended to have lower RTS and P_s_, indicating more severe physiological derangement and a lower probability of survival. Additionally, the proportion of patients presenting with hemorrhagic shock on hospital arrival was markedly higher in the early-death group versus the survivor group. These findings suggest that early deaths in the short TPT group reflect severe physiological compromise already present at the time of hospital arrival.

In the logistic regression analysis, ISS was significantly associated with early mortality, whereas TPT was not independently associated with early mortality after adjustment for ISS. These findings suggest that early deaths in the short TPT group were more closely related to injury severity than to prehospital time itself. Because the study included patients with associated injuries, such as TBI, early mortality cannot be explained solely by hemorrhagic shock and likely reflects overall injury severity.

Recent analyses using the North American National Emergency Medical Services Information System database have shown that prehospital time is shortest in urban areas and progressively longer in suburban, rural, and wilderness areas among trauma patients at risk of hemorrhagic shock [35]. These findings suggest that prehospital time is influenced not only by patient condition but also by geographic and EMS system factors. In urban trauma systems, hemorrhage is the most common cause of preventable trauma death, and many of these deaths occur in the prehospital phase or within the first hour after hospital arrival [36]. Taken together, these findings suggest that the observed association between shorter TPT and early mortality is more likely explained by confounding factors inherent to urban EMS systems rather than a harmful effect of shorter TPT itself.

In densely populated urban areas, severely injured patients are more likely to be transported rapidly. Several factors specific to urban environments may contribute to this phenomenon. First, traffic accidents are more likely to be witnessed, resulting in rapid emergency activation. Second, the geographic distance between ECCCs is relatively short, allowing for rapid hospital transport. Additionally, destination hospital selection is often initiated at the time of emergency call based on initial triage information and patient condition. These transport patterns may partially explain the higher proportion of early deaths observed in the short TPT group.

To evaluate physiological deterioration during transport, we performed an ISS-stratified analysis using DSI with a cutoff value of 0.1. The proportion of DSI-positive cases was higher in the group with ISS of 25–49 and shorter TPT; however, DSI did not clearly distinguish between survivors and non-survivors. In this urban trauma system, where TPT is relatively short, a single indicator, such as DSI, may not adequately capture physiological deterioration during transport. Additionally, the small number of patients in the group with ISS ≥ 50 may have affected the statistical power of the analysis.

Furthermore, the proportion of DOA cases was higher in the shortest TPT group, whereas the proportion of DSI-positive cases was higher in the longer TPT group. These findings suggest that, in urban trauma systems, severely injured patients may reach the hospital before cardiac arrest when TPT is short, whereas patients with longer TPT may deteriorate during transport. This pattern supports the interpretation that early deaths in the short TPT group reflect injury severity at the time of injury rather than physiological deterioration during transport.

Our results are consistent with previous studies suggesting that shorter prehospital time may be associated with higher mortality, particularly in urban settings and among patients with torso trauma [25–28]. Studies on the timing of trauma death have also suggested that early deaths after hospital arrival may share characteristics with deaths occurring immediately after injury in severely injured patients [37]. Together with our findings, these results suggest that, in urban trauma systems, rapid transport may shift the location of death from the scene or during transport to the hospital, and overall survival may not necessarily improve uniformly. Therefore, the observed association between shorter TPT and early mortality likely reflects the structure of urban EMS systems and injury severity rather than the effect of prehospital time itself.

One of the key strengths of this study is that it could identify injury locations using prehospital EMS data and analyze the geographic distribution in relation to injury severity. GIS analysis showed that early deaths were concentrated within a relatively short distance from the study center, whereas patients transported from longer distances tended to have a lower proportion of severe injuries. This pattern likely reflects the structure of urban EMS system in Tokyo, where multiple ECCCs are located within a relatively small geographic area. Consequently, more severely injured patients are more likely to be transported to the nearest center, resulting in a concentration of severely injured patients in the short-distance transport group.

This study provides new insights into the relationship between TPT and early mortality in urban trauma systems by focusing on severe torso trauma and incorporating GIS-based spatial analysis. These findings further clarify the geographic distribution of injury severity and outcomes in an urban EMS system.

In urban trauma systems where prehospital times are already relatively short, further reduction in TPT alone may not necessarily lead to improved outcomes. Instead, strengthening early in-hospital management for severely injured patients may be more important. Such efforts may include rapid trauma team activation, early surgical or interventional hemorrhage control, and prompt initiation of resuscitative care.

### Limitations and future directions

This study has some limitations. First, this was a single-center retrospective observational study with a relatively small sample size, which limits the generalizability of the findings. Additionally, selection bias and residual confounding cannot be completely excluded.

Second, the strict exclusion criteria may have narrowed the study population. Patients who received advanced on-site interventions by DMAT and those in whom hemorrhagic shock was not considered the primary cause of death were excluded to minimize confounding when evaluating the association between prehospital time and outcomes. However, these exclusions may also have led to underrepresentation of severely injured patients and limited the generalizability of the findings. Additionally, data on the interval between injury and EMS activation was not available.

Third, ISS appeared relatively low even among deceased patients. One possible explanation is that patients with extremely severe injuries frequently experienced cardiopulmonary arrest at the scene and were therefore excluded according to the study criteria. Consequently, cases with the highest anatomical severity may have been systematically excluded, which may partly explain the relatively low ISS observed in the overall cohort and among non-survivors.

Fourth, because we included only patients transported to our institution, outcome data for patients transported to other hospitals within the same medical care zone were unavailable. Therefore, we could not directly evaluate whether the most critically injured patients were preferentially transported to the nearest facility. This limitation reflects the structure of regional trauma system and lack of linked outcome data across hospitals in Japan.

Fifth, approximately 90% of the study population had blunt trauma, reflecting the epidemiology of trauma in Japan. Additionally, a substantial proportion of patients had concomitant TBI associated with traffic accidents or falls. Therefore, the findings are most directly applicable to blunt severe torso trauma in an urban Japanese trauma system, and caution is required when generalizing these results to penetrating trauma or other trauma populations.

Sixth, because the number of early deaths was limited (*n* = 20), multivariable analysis was restricted to a parsimonious model adjusted only for ISS. Consequently, other potentially important confounders, such as age, mechanism of injury, and concomitant TBI, could not be fully adjusted for.

Finally, several measurement-related limitations should be acknowledged. Survival probability was assessed using TRISS-derived P_s_, which remains widely used but may underestimate survival probability in contemporary trauma systems.

Transport distance was estimated using Manhattan distance based on geographic coordinates and therefore did not reflect the actual ambulance route, traffic conditions, signal control, or emergency vehicle operation. However, because TPT was analyzed using actual measured values for each patient rather than travel time estimated from distance, distance was used only as a supplementary indicator of the geographic characteristics of incident locations.

Further multicenter studies with larger sample sizes and region-wide data linkage are warranted to validate these findings and to clarify how urban trauma system structure influences the relationship between prehospital time and early mortality.

## Conclusions

In this study, shorter TPT was associated with early mortality among patients with severe torso trauma transported to an urban ECCC. This finding likely reflects the rapid transport of critically injured patients rather than a harmful effect of shorter TPT itself.

In urban trauma systems where prehospital transport times are already relatively short, the observed association between shorter TPT and early mortality should be interpreted in the context of patient severity and trauma system structure. In this context, optimizing early in-hospital response may also be important for improving outcomes in critically injured patients. Further studies are needed to clarify how prehospital time and early in-hospital care interact to influence outcomes in severely injured patients.

## Supplementary Information

Below is the link to the electronic supplementary material.

Supplementary Material 1

## Data Availability

The data used in this study were extracted from electronic medical records at a single institution. Due to privacy regulations and institutional policies, these data are not publicly available.

## Abbreviations

AIS: Abbreviated Injury Scale
CI: Confidence interval
DMAT: Disaster medical assistance team
DOA: Dead on arrival
DSI: Delta Shock Index
ECCC: Emergency and critical care center
ED: Emergency department
EMS: Emergency medical service
GCS: Glasgow Coma Scale
GIS: Geographic information system
IQR: Interquartile range
ISS: Injury Severity Score
JTDB: Japan Trauma Data Bank
OR: Odds ratio
OST: On-scene time
P_s_: Probability of survival
RT: Response time
RTS: Revised Trauma Score
SI: Shock index
TBI: traumatic brain injury
TPT: Total prehospital time
TRISS: Trauma and Injury Severity Score
TT: Transport time
