# Supplementary material for: Association between total prehospital time and early mortality in patients with severe torso trauma: a retrospective study at an urban Emergency and Critical Care Center
Source: Int J Emerg Med. 2026 May 8;19:166. doi: 10.1186/s12245-026-01249-3 (PMC13321564; doi:10.1186/s12245-026-01249-3)
Supplement: Supplementary file 1 — Supplementary Material 1 [file 12245_2026_1249_MOESM1_ESM.docx]

**Additional File 1**

**Supplemental Methods**

1. Study setting

This study was conducted at a single university hospital located in a secondary medical care zone in the northeastern metropolitan part of Tokyo. As of March 2024, this zone comprised three administrative districts, with a population of approximately 1.37 million and a total area of 98 km^2^. The study site is the only tertiary care institution and Emergency and Critical Care Center (ECCC) in the designated zone.

1. Emergency and Critical Care Center

The ECCC in Japan is a tertiary emergency care facility that provides 24-hour advanced emergency and critical care for patients with severe trauma, cardiac arrest, stroke, acute coronary syndrome, and other life-threatening conditions. ECCCs are designated by the government and serve as regional tertiary emergency care centers.

The study hospital functions as the only ECCC in the designated secondary medical care zone and is responsible for receiving severely ill and injured patients from this area. The center provides comprehensive trauma care, including trauma team activation, emergency surgery, damage control surgery, interventional radiology, and intensive care management.

Because the center is the only tertiary emergency facility in this medical zone, most patients with severe trauma in the area are transported to this hospital.

1. Tokyo Emergency Medical Services system

The Tokyo prehospital emergency medical service (EMS) system is a fire department–based ambulance system operated by the Tokyo Fire Department, which centrally coordinates all EMS dispatches. All emergency transports are conducted via ambulance, and each ambulance crew includes at least one nationally certified paramedic. Under online medical control, paramedics are authorized to perform advanced procedures such as intravenous access, airway management, and administration of selected emergency medications.

Hospital selection is based on triage criteria tailored to each type of injury or illness, and patients are transported to the most appropriate nearby hospital based on injury severity and hospital acceptance. In cases of severe trauma, the nearest available ECCC is prioritized based on the geographic location of the incident, regardless of medical administrative boundaries.

Although intravenous fluid administration is indicated for trauma-related shock, it is seldom performed in severe trauma cases because rapid transport is prioritized. Physician-staffed response units, such as doctor cars, operate in limited areas but are not routinely involved in trauma care. Prehospital blood transfusion is not performed in Tokyo.

Because the EMS system operates in a densely populated urban environment, response and transport times are generally short. As of 2024, the average response time (RT) was 8 min and 59 s.

1. Trauma system structure in Tokyo

In contrast to centralized trauma systems, such as those in the United States, where severely injured patients are concentrated in a limited number of trauma centers, trauma patients in Tokyo are distributed among multiple ECCCs across the metropolitan area. Although our center receives a large number of emergency patients overall, trauma cases represent only a subset of these patients.

1. Disaster Medical Assistance Team

In Japan, the Disaster Medical Assistance Team (DMAT) is part of a national disaster medical response system designed for rapid deployment to disaster areas to provide on-site triage, initial medical care, coordination of patient transport, and medical care at disaster-affected hospitals during disasters and mass-casualty incidents. In addition to disaster response, regional DMAT teams may also be dispatched to severe trauma scenes requiring complex rescue operations, such as entrapment or prolonged extrication. In such cases, DMAT personnel may perform medical interventions at the scene before transport.

Because patients who receive on-scene medical interventions by DMAT may have substantially different prehospital time structures and medical management compared with standard EMS transport, these patients were excluded from this study to maintain a more homogeneous study population and minimize confounding related to on-scene medical interventions.

6. TRISS methodology

Additionally, TRISS was used to estimate survival probability as it is the standard prognostic score adopted in JTDB. Although more advanced prognostic models, such as Revised Injury Severity Classification (RISC) and RISC II, have been proposed, they require additional physiological and laboratory variables not collected in JTDB. Therefore, TRISS was considered the most practical prognostic score for consistently estimating survival probability across all cases in this registry-based study.
